# The Odontographic Society of Cincinnati

**Published:** 1897-04

**Authors:** 


### The Odontographic Society of Cincinnati.

The regular monthly meeting of this Society was held Friday evening, March 26th, at the office of Dr. H. F. Vandervoort, in the Berkshire Building. Nearly all the active members were present. An interesting paper on orthodontia was read by Dr. F. Sage, which contained some valuable suggestions in regard to treatment, and raised some interesting questions in regard to some of the present modes of treatment. The subject was pretty fully discussed by Drs. Hunter, H. T. Smith, Callahan, Lock, and others.

While this society does not strive to do great things, yet it has been and is a great benefit to the dental profession of Cincinnati. Long may it live and prosper.

